# Multi-stage optimization of a deep model: A case study on ground motion modeling

**DOI:** 10.1371/journal.pone.0203829

**Published:** 2018-09-19

**Authors:** Amirhessam Tahmassebi, Amir H. Gandomi, Simon Fong, Anke Meyer-Baese, Simon Y. Foo

**Affiliations:** 1 Department of Scientific Computing, Florida State University, Tallahassee, Florida 32306-4120, United States of America; 2 School of Business, Stevens Institute of Technology, Hoboken, New Jersey 07030, United States of America; 3 Department of Computer Science and Information Science, University of Macau, Taipa, Macau; 4 Department of Electrical and Computer Engineering, FAMU-FSU College of Engineering, Tallahassee, Florida 32310-6046, United States of America; Liverpool John Moores University, UNITED KINGDOM

## Abstract

In this study, a multi-stage optimization procedure is proposed to develop deep neural network models which results in a powerful deep learning pipeline called intelligent deep learning (iDeepLe). The proposed pipeline is then evaluated by a challenging real-world problem, the modeling of the spectral acceleration experienced by a particle during earthquakes. This approach has three main stages to optimize the deep model topology, the hyper-parameters, and its performance, respectively. This pipeline optimizes the deep model via adaptive learning rate optimization algorithms for both accuracy and complexity in multiple stages, while simultaneously solving the unknown parameters of the regression model. Among the seven adaptive learning rate optimization algorithms, Nadam optimization algorithm has shown the best performance results in the current study. The proposed approach is shown to be a suitable tool to generate solid models for this complex real-world system. The results also show that the parallel pipeline of iDeepLe has the capacity to handle big data problems as well.

## Introduction

Development of the first computational model based on artificial neural networks (ANN) with application to artificial intelligence (AI) might date back to a model built in 1943, which was inspired from biology to simulate how the brain works [1]. Neurons in the perceptual system represent features of the sensory input. The brain has a deep architecture and learns to extract many layers of features, where features in one layer represent combinations of simpler features in the layer below and so on. This is referred to as feature hierarchy. Based on this idea, several architectures for the topology of the networks such as layers of neurons with fully/sparse connected hidden layers were proposed. Two essential questions to ask are: How can the weights that connect the neurons in each layer be adjusted? How many parameters should we find and how much data is necessary to train or test the network?

Shallow neural networks were the models before 2006 due to the failure of deep neural networks at training/testing data due to the lack of computational equipment. The revolution in deep neural networks began with Hinton’s Deep Belief Networks (DBN) based on Restricted Boltzmann Machines (RBM) [2] as well as Bengio’s Greedy Layer-Wise Training of Deep Networks [3]. This was a breakthrough in learning deep architectures using different perspectives of optimization. Deep learning models involve optimization from the first day of appearance of neural networks. For example, [4] proposed Support Vector Machines (SVM) just by employing a smart optimization algorithm in implementing the single layer perceptron, which is still one of the best methods implemented in machine learning. This highlights the importance of the optimization algorithms in development of the deep neural topologies. Deep learning explores complicated structures especially in big data problems with the help of the backpropagation optimization algorithm to compute the hyper-parameters involved in the network [5].

Emerging as one of the most contemporary machine learning techniques, deep learning has shown success in areas such as image classification, speech recognition, and even playing games through the use of hierarchical architecture which includes many layers of non-linear information [6] [7] [8]. Availability of huge amounts of training data, powerful computational infrastructure, and advances in academia could be named as the three main bedrocks of recent deep learning success. This encourages the development of deep learning models to be applied to real-world problems. Importantly, we live in an era where we have sufficient computational equipment and cutting-edge technologies that allow us to better optimize the hyper-parameters involved in deep neural networks. However, developing new deep models for classification or regression tasks to solve real-world problems still demands robust optimization techniques [9] [10]. It should be noted that, at this point, there is no consensus on choosing the right optimization method based on the network topology and architecture [6].

One of the most famous projects in the ground motion modeling is the Next Generation Attenuation of Ground Motion (NGA) [11] model which tries to formulate the experienced particles (e.g. buildings) motions during earthquake. The NGA project is a multidisciplinary research program coordinated by the Lifelines Program of the Pacific Earthquake Engineering Research Center (PEER) (http://peer.berkeley.edu/), in partnership with the U.S. Geological Survey (https://earthquake.usgs.gov/) and the Southern California Earthquake Center (https://scec.org/) for purposes of seismic hazards assessment. For the NGA project, a comprehensive database of strong ground-motions assembled by PEER [12].

In this paper, a powerful pipeline is developed in order to improve the NGA using deep neural networks to predict spectral acceleration with continuous values based on the ground-motion variables such as ground and earthquake parameters. The deep model is optimized in multiple stages: 1) finding the most efficient topology of the network in terms of the number of layers, number of neurons in each layer, and the activation function for each layer, 2) the learning rates for each optimization methods, and 3) optimization of metric scores to see the performance of the network. At last, the results are compared and validated with one of the available famous NGA models [11] and some other machine learning approaches.

## Deep learning model

**Fig 1:**
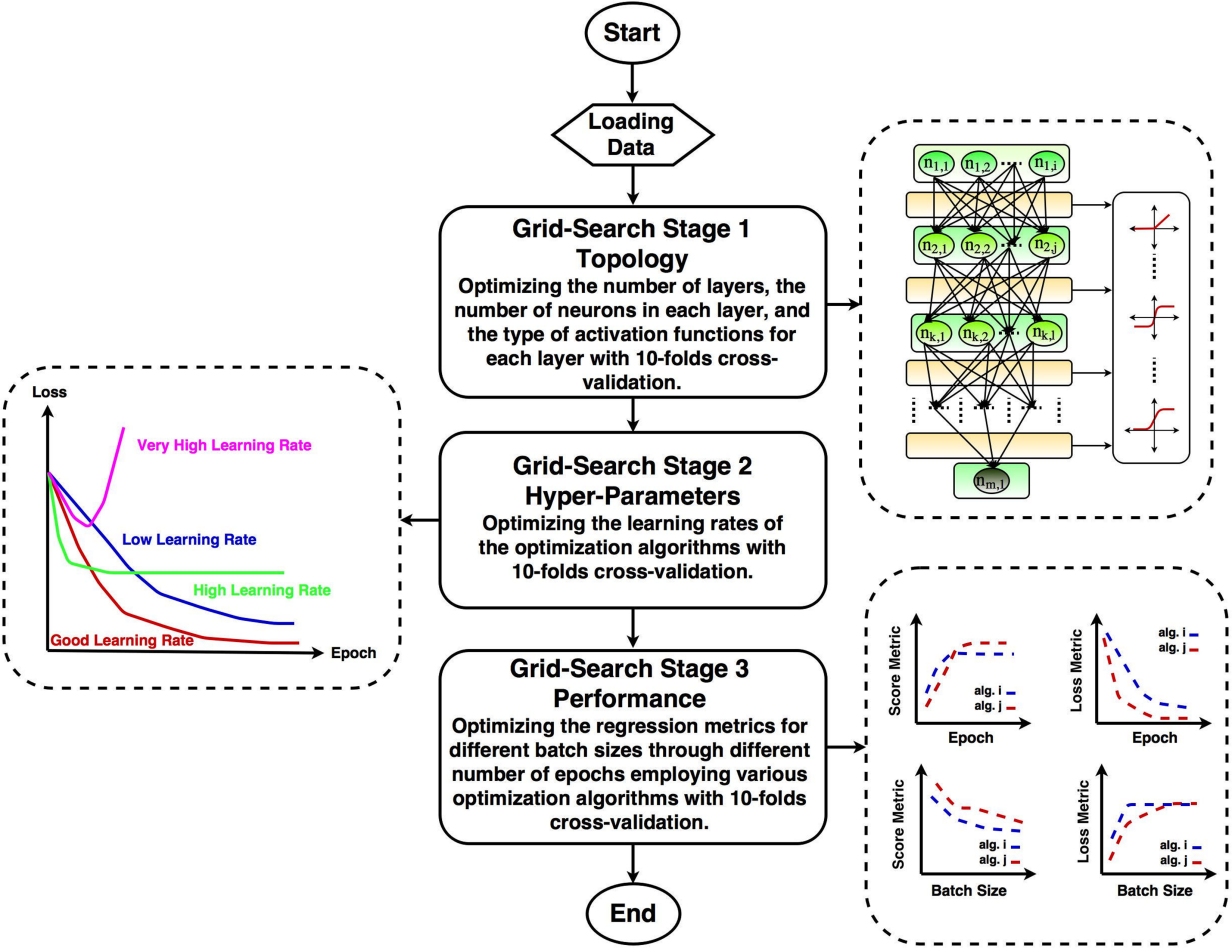
iDeepLe flowchart.

In this section the proposed pipeline is introduced which consists multiple stages as shown in Fig 1. In this pipeline, numerous layers of neurons inspired by sequential modeling were stacked on each other to construct the core data structure of iDeepLe [13]. Each layer by itself likewise the other neural layers contains cost function, learning rate, activation function, and also some regularization layouts based on the employed optimization algorithm. The important part and novelty of iDeepLe is that each layer in the sequential deep model can perform independently as a module with minimum finitudes. In principle, each layer can interact with the other layers and does not limit the performance of the other layers. The proposed pipeline, iDeepLe is written in Python with the help of various API libraries such as Keras [14], TensorFlow [15], and Scikit-Learn [16] [17]. In the modeling architecture of iDeepLe, all the benefits of the aforementioned API libraries have been combined to maximize the performance. In addition, due to the dynamic structure of iDeepLe, it is also capable of doing classification tasks.

As previously discussed, each model is a sequence of different layers and each layer is a fully configurable module which requires different hyper-parameters including learning rate, stopping criteria, batch size, number of epochs, and regularization rates. The arrangement of hyper-parameter settings affects the performance of training phase of the model. For example, the learning rate (step size) indicates how far we should step against the direction which has the steepest rate of increase based on the gradient of the cost function. In addition to this, computing the loss function over the entire training data, especially in cases dealing with large-scale data would be costly. In this regard, employing a smart approach to compute the gradient over batches of the training data and updating the weights would help us overcome this challenge. Furthermore, the core idea of a deep learning task is finding the weights through iterative refinement. This means the number of epochs or iterations would also affect the stability and performance of the network. Thus, at this point, the essential questions to be answered are: 1) How can we design the topology of the networks in terms of width and depth? 2) What should we choose as the learning rate, batch size, and number of epochs? 3) What is the best way to optimize the cost function?

To encounter all of the aforementioned challenges and meet a reasonable confidence interval for the results, we employed an exhaustive grid-search with cross-validation in three different stages across all of the hyper-parameters to find the optimal performance. It should be noted that each layer of the network requires a huge number of operations due to the various number of tuned hyper-parameters and this would be further multiplied with the cost of the other layers too. This would suggest writing the code using parallel algorithms and employing high performance computing (HPC) and GPU-enabled machines overcome this challenge. However, we have proposed a smart randomized search model as the second option in iDeepLe for the people who do not have access to HPC machines. The grid-search can be done across the number of layers, the number of neurons in each layer, the types of activation functions, the types of cost functions, the different learning rates, the number of batch sizes, the number of epochs, and the types of optimization algorithms. In general, the design of the topology of the network and the choice of optimization algorithm seem to depend principally on the user’s acquaintance with different types of neural networks and their knowledge about optimization algorithms in terms of tunning the aforementioned hyper-parameters [6]. However, [18] have previously compared the performance of several optimization algorithms in various learning tasks which suggested to us that some of these algorithms should be considered. Additionally, it should be noted that to reduce the possibility of over-fitting through the optimization process, for each cross-validation, a stochastic random seed based on Numpy library was used to generate a non-biased fold of data. This process has been done for each three stages separately. In better words, the process of the finding of the best hyper-parameters for this problem is totally data-independent.

In this pipeline, the exhaustive grid-search is wisely splitted into three stages with considering different constraints. Fig 1 demonstrates the flowchart of the proposed pipeline. In the first stage, it is desired to optimize the topology of the network to find the number of layers, the number of neurons in each layer, and the type of activation function for each layer. As the goal of the second stage, the optimization of the learning rates for each of the optimization algorithms is desired. Lastly, in the third stage, the performance of the model with the optimized topology and learning rates is tested with different number of epochs and batch sizes for different optimization algorithms. In this regard, the regression scores and losses are chosen as the performance metrics.

As discussed, after loading the training data set, it is desired to optimize the topology of the deep neural networks in terms of the number layers, the number of neurons in each layer, and also the type of activation function which should be considered for each layer. In this regard, a grid-search employing 10-folds cross-validation with fixed values for batch size, number of epochs, and optimization algorithm was considered. Table 1 presents the details of the hyper-parameters that were set for the optimization of the topology in the grid-search stage 1. Due to the large number of operations, the parallel code ran on 20 nodes of HPC machines at FSU Research Computing Center (RCC) (https://rcc.fsu.edu/). In addition, to accelerate the grid-search process and ensure the stability of the network, some constraints were considered. First, to optimize the numbers of neurons in the hidden layers, it is assumed that the number of neurons in the lower layers cannot be more than the number of neurons in the top layers. For example, if the layer two has 20 neurons, the number of neurons in the layer three should be less than 20. It decreases the number of operations drastically (∼*O*(*n*^2^)). For example, the total computation time of the optimized architecture which contains 1962 trainable parameters using a batch size of 50 through 1000 epochs was 287 seconds and the whole parallel calculation using 10-20 HPC nodes took ∼36 hours. In addition to this, since the metric which is desired in this problem is the coefficient of determination and also the output, *Ln*(*SA*) was normalized to have values between 0 and 1, one neuron was always considered in the last layer with Sigmoid as the activation function. In this way, the model would always produce one output which is between 0 and 1.

**Table 1:** Parameters setting for the grid-search stage 1.

Hyper-parameter	Settings
Number of layers	3, 4, 5, 6, 7, 8, 9, 10, 12, 15, 20
Number of neurons in each layer	1, 2, 3, 4, 5, 6, 7, 8, 9, 10, 12, 15, 20, 25, 30, 35, 40, 45, 50
Batch sizes	50
Number of epochs	1000
Activation functions	ReLU, Tanh, Softplus, Softsign, Linear, Softmax, Sigmoid
Optimizers	Adam
Learning rates	0.001
Losses	MSE
Score metrics	R
Number of HPC nodes	20
Number of folds in cross-validation	10

**Fig 2:**
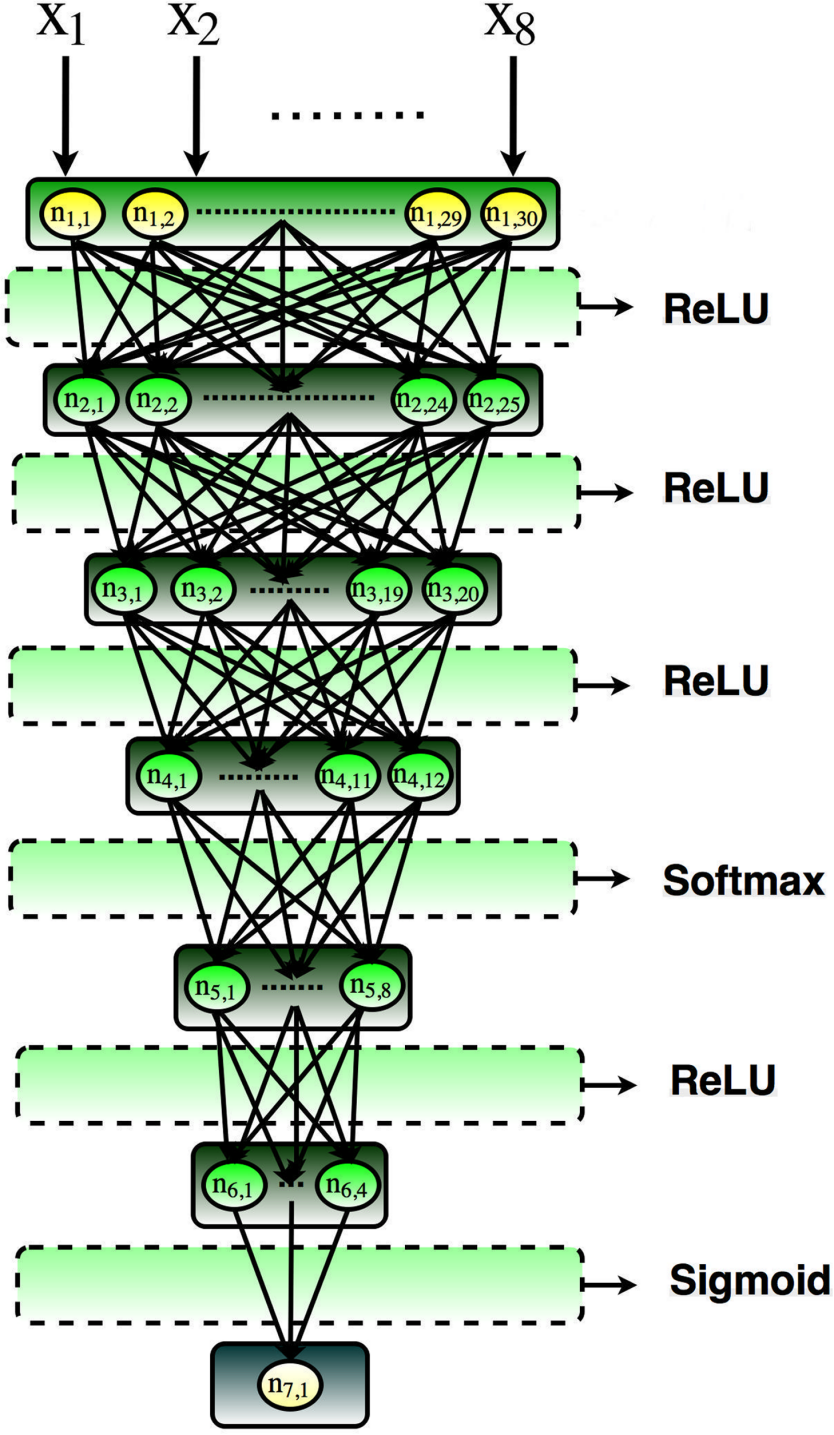
The architecture of the optimized deep neural network model.

The architecture of the optimized model after the grid-search stage 1 is presented in Fig 2. It contains 8 layers and each layer has 8, 30, 25, 20, 12, 8, 4, 1 neurons, respectively. The number of trainable parameters for each layer (starting from the first layer to the last layer) are 270, 775, 520, 252, 104, 36, and 5 with a total number of 1962 parameters. Each layer also requires an activation function. In this deep architecture, Rectified Linear Unit (ReLU) [19], Softmax [20], and Sigmoid [21] have been employed as the activation functions. The properties of the activation functions used in different layers of the deep model are also discussed in Table 2. In optimization section, we will discuss various numbers of optimization algorithms with the hope of increasing the user’s familiarity with the optimization algorithms.

**Table 2:** Properties of the activation functions used in different layers of iDeepLe.

Function	Equation	Derivative	Range
ReLU	$f\left(x\right)=\{\begin{array}{cc} 0 & \text{for}\;x<0\\ x & \text{for}\;x\geq 0 \end{array}$	$f^{\prime }\left(x\right)=\{\begin{array}{cc} 0 & \text{for}\;x<0\\ 1 & \text{for}\;x\geq 0 \end{array}$	[0, ∞)
Softmax	$f_{i}\left(\vec{x}\right)=\frac{\exp(x_{i})}{\sum_{j=1}^{n}\exp\left(x_{j}\right)}$	$\frac{\partial f_{i}\left(\vec{x}\right)}{\partial x_{j}}=f_{i}\left(\vec{x}\right)\left(\delta_{ij}-f_{j}\left(\vec{x}\right)\right)$	(0, 1)
Sigmoid	$f\left(x\right)=\frac{1}{1+\exp(-x)}$	$f^{\prime }\left(x\right)=\frac{\exp(-x)}{{\left[1+\exp\left(-x\right)\right]}^{2}}$	(0, 1)

## Optimization

Deep learning algorithms involve optimization in many contexts. The core idea of optimization is finding the best weights *w* at each layer of the topology of the deep neural networks with the hope that these weights increase the performance metric (decreasing cost) on the entire training set [6]. It is clear that the whole procedure, similar to other machine learning tasks, is indirect and the true distribution of the training data set is unknown. This is in contrast to the traditional optimization algorithms in which optimizing the pure function is the direct goal. Assume the true distribution of the training data set is known and *f*(*x*) as well, which turns into a simple pure optimization problem. To address this problem and find a way to convert the machine learning task back into the pure optimization problem, the unknown true distribution of the training data was substituted with the empirical distribution defined by the training data and then the optimization algorithms were applied [6] [22].

Optimization by itself is an arduous and time-consuming problem. This general problem becomes even more challenging once we are dealing with non-convex problems such as deep neural networks. In traditional machine learning algorithms, it is most common to set up the objective function and constraints to avoid solving the general non-convex optimization problems [23] [20]. Even in solving convex optimization problems, difficulties such as ill-conditioning, local minima, saddle points, and steep cliff structures in gradients may be encountered and introduce further challenges. To emphasize the aforementioned challenges, we have employed seven optimization algorithms including basic algorithms and algorithms with adaptive learning rates for the training of the deep models. Additionally, we have explained the pros and cons of the pseudo codes using each as the optimization algorithm for training the deep neural networks.

### Stochastic gradient descent (SGD)

Employing gradient descent and its variants as the optimization algorithms for deep learning and other machine learning tasks is probably the most frequent choice among users. Based on the amount of the training data we assign to compute the gradient of the objective function, there will be three different variants of gradient descent [24]. The first variant is vanilla gradient descent, also known as batch gradient descent. Here, the entire training data set is used for calculation of the gradient of the cost function with respect to the weights. This process is fairly slow and requires only one update due to the use of the entire data set (one update per epoch). The second variant is stochastic gradient descent which performs an update for each of the training instances which leads to redundant computational operations for fairly large data sets. Finally, the third and also the popular variant, is mini-batch gradient descent which performs an update for every mini-batch of size *m* training instances. It should be noted that mini-batch gradient descent is the typical algorithm for training neural networks. The term SGD is usually employed when the mini-batch gradient descent algorithm is used (like in this paper) [6] [24]. The pseudo code for SGD is shown in Algorithm 1.

**Algorithm 1:** SGD

**Input:** Training data *S*, learning rate *η*, weights *w*,

**Output:** Updated weights *w*

**1** *w* ← *w*_0_;

**2** **while** *stopping criterion is not met* **do**

**3**  Randomly shuffle the training data *S*;

**4**  Sample a minibatch of size *m*:{(*x*^(1)^, *y*^(1)^), …, (*x*^(*m*)^, *y*^(*m*)^)} ∈ *S*;

**5**  **for** *i* ∈ {1, …, *m*} **do**

**6**   $\hat{G}\leftarrow \frac{\partial }{\partial w_{i}}cost\left(w,\left(x^{\left(i\right)},y^{\left(i\right)}\right)\right)$; Gradient calculation

**7**  **end**

**8**  $w\leftarrow w-\eta \hat{G}$;

**9** **end**

### Adaptive gradient (Adagrad)

Adagrad is another variant of gradient based optimization algorithms which has the ability of adapting the learning rate based on the data characteristics at each iteration. It assigns lower learning rates to the repetitive features and also higher learning rates to less repetitive features. In this way, rare but possible features are being detected automatically. Algorithm 2 explains the pseudo code for updating the parameters at each iteration. Applying the Adagrad algorithm to any sparse training data set would show the high performance of this algorithm in prediction of infrequent features in the training data set by adapting the learning rates dynamically [25]. It would also outperform slow algorithms such as vanilla gradient descent once we are dealing with big data problems. Conversely, the drawback is zero convergence for long iterations [6] [24].

**Algorithm 2:** Adagrad

**Input:** Training data *S*, learning rate *η*, weights *w*, fuzz factor *ϵ*, learning rate decay over each update *r*

**Output:** Updated weights *w*

**1** *ϵ* ← *ϵ*_0_ ≈ 10^−8^;

**2** *w* ← *w*_0_;

**3** *r* ← 0;

**4** **while** *stopping criterion is not met* **do**

**5**  Randomly shuffle the training data *S*;

**6**  Sample a minibatch of size *m*:{(*x*^(1)^, *y*^(1)^), …, (*x*^(*m*)^, *y*^(*m*)^)} ∈ *S*;

**7**  **for** *i* ∈ {1, …, *m*} **do**

**8**   $\hat{G}\leftarrow \frac{\partial }{\partial w_{i}}cost\left(w,\left(x^{\left(i\right)},y^{\left(i\right)}\right)\right);$ Gradient calculation

**9**  **end**

**10**  $r\leftarrow r+\hat{G}⊙\hat{G};$

**11**  $w\leftarrow w-\frac{\eta }{\epsilon +\sqrt{r}}⊙\hat{G};$

**12** **end**

### Adaptive delta (Adadelta)

As explained in section 9, as we increase the number of epochs for longer iterations, the learning rate fails to converge to zero. To improve this, two main ideas are needed. The first idea is restricting the past gradients for a fixed size instead of incorporating the whole historical gradient information. This approach scales the learning rate and avoids observing discontinuity in the learning progress. The second idea is employing an acceleration term such as momentum to process the first idea. Contradictory to the Adagrad algorithm, the Adadelta is insensitive to hyper-parameters [26].

**Algorithm 3:** Adadelta

**Input:** Training data *S*, learning rate *η*, weights *w*, decay rate *ρ*, fuzz factor *ϵ*

**Output:** Updated weights *w*

**1** *ρ* ← *ρ*_0_;

**2** *ϵ* ← *ϵ*_0_ ≈ 10^−8^;

**3** *w* ← *w*_0_;

**4** $E{\left[{\hat{G}}^{2}\right]}_{t=0}\leftarrow 0;$

**5** *E*[Δ*w*^2^]_*t*=0_ ← 0;

**6** **for** *t* ∈ {1, …, *T*} **do**

**7**  Randomly shuffle the training data *S*;

**8**  Sample a minibatch of size *m*:{(*x*^(1)^, *y*^(1)^), …, (*x*^(*m*)^, *y*^(*m*)^)} ∈ *S*;

**9**  **for** *i* ∈ {1, …, *m*} **do**

**10**   ${\hat{G}}_{t}\leftarrow \frac{\partial }{\partial w_{i}}cost\left(w_{t},\left(x^{\left(i\right)},y^{\left(i\right)}\right)\right);$ Gradient calculation

**11**  **end**

**12**  $E{\left[{\hat{G}}^{2}\right]}_{t}\leftarrow \rho E{\left[{\hat{G}}^{2}\right]}_{t-1}+\left(1-\rho \right){{\hat{G}}_{t}}^{2};$

**13**  ${\Delta w}_{t}\leftarrow -\frac{\sqrt{E{\left[\Delta w^{2}\right]}_{t-1}+\epsilon }}{\sqrt{E{\left[{\hat{G}}^{2}\right]}_{t}+\epsilon }}{\hat{G}}_{t};$

**14**  *E*[Δ*w*^2^]_*t*_ ← *ρE*[Δ*w*^2^]_*t*−1_ + (1 + *ρ*)Δ*w*^2^_*t*_;

**15**  *w*_*t*+1_ ← *w*_*t*_ + Δ*w*_*t*_;

**16** **end**

### Adaptive moment estimation (Adam)

This algorithm is presented as a generalization of the Adagrad algorithm by calculating and updating some statistics such as the first and the second moments of historical gradients at each iteration. In this regard, it does require a little memory to process [6]. With the help of these two features, Adam is employed for the big data problems in terms of both dimension and volume. Like the Adagrad algorithm, Adam is a smart choice for training data with sparse and noisy gradients [24]. In addition to this, Adam also works for non-stationary objective functions since it converges due to the change of objective function during the iterations automatically [27]. In fact, Adam carries the benefits of both Adagrad and RMSprop algorithms by assigning the decay rates *β*_1_ and *β*_2_ to the exponential moving average of the gradient and its square.

**Algorithm 4:** Adam

**Input:** Training data *S*, learning rate *η*, weights *w*, fuzz factor *ϵ*, learning rates decay over each update *r*_1_ and *r*_2_, exponential decay rates *β*_1_ and *β*_2_

**Output:** Updated weights *w*

**1** *ϵ* ← *ϵ*_0_ ≈ 10^−8^;

**2** *w* ← *w*_0_;

**3** *r*_1_ ← 0;

**4** *r*_2_ ← 0;

**5** *t* ← 0;

**6** **while** *stopping criterion is not met* **do**

**7**  Randomly shuffle the training data *S*;

**8**  Sample a minibatch of size *m*:{(*x*^(1)^, *y*^(1)^), …, (*x*^(*m*)^, *y*^(*m*)^)} ∈ *S*;

**9**  **for** *i* ∈ {1, …, *m*} **do**

**10**   $\hat{G}\leftarrow \frac{\partial }{\partial w_{i}}cost\left(w,\left(x^{\left(i\right)},y^{\left(i\right)}\right)\right);$ Gradient calculation

**11**   *t* ← *t* + 1;

**12**  **end**

**13**  $r_{1}\leftarrow \beta_{1}r_{1}+\left(1-\beta_{1}\right)\hat{G};$

**14**  $r_{2}\leftarrow \beta_{2}r_{2}+\left(1-\beta_{2}\right)\hat{G}⊙\hat{G};$

**15**  $\hat{r_{1}}\leftarrow \frac{r_{1}}{1-\beta_{1}^{t}};$

**16**  $\hat{r_{2}}\leftarrow \frac{r_{2}}{1-\beta_{2}^{t}};$

**17**  $w\leftarrow w-\eta \frac{\hat{r_{1}}}{\epsilon +\sqrt{\hat{r_{2}}}};$

**18** **end**

### Adaptive moment estimation based on the infinity norm (Adamax)

As it is shown in Algorithm 4, the weights are updated based on the *L*_2_ norm of their previous and current gradients. This approach can be generalized by considering the *L*_∞_ norm instead of the *L*_2_ norm. In other words, Adamax is a variant of Adam based on the infinity norm. The details of the Adamax algorithm have been explained in Algorithm 1.

**Algorithm 5:** Adamax

**Input:** Training data *S*, learning rate *η*, weights *w*, fuzz factor *ϵ*, learning rate decay over each update *r*, exponentially weighted infinity norm *u*, exponential decay rates *β*_1_ and *β*_2_

**Output:** Updated weights *w*

**1** *ϵ* ← *ϵ*_0_ ≈ 10^−8^;

**2** *w* ← *w*_0_;

**3** *r* ← 0;

**4** *u* ← 0;

**5** *t* ← 0;

**6** **while** *stopping criterion is not met* **do**

**7**  Randomly shuffle the training data *S*;

**8**  Sample a minibatch of size *m*:{(*x*^(1)^, *y*^(1)^), …, (*x*^(*m*)^, *y*^(*m*)^)} ∈ *S*;

**9**  **for** *i* ∈ {1, …, *m*} **do**

**10**   ${\hat{G}}_{t}\leftarrow \frac{\partial }{\partial w_{i}}cost\left(w_{t},\left(x^{\left(i\right)},y^{\left(i\right)}\right)\right);$ Gradient calculation

**11**   *t* ← *t* + 1;

**12**  **end**

**13**  $r_{t}\leftarrow \beta_{1}r_{t-1}+\left(1-\beta_{1}\right){\hat{G}}_{t};$

**14**  $u_{t}\leftarrow \max(\beta_{2}u_{t-1},|{\hat{G}}_{t}\left|\right);$

**15**  $w_{t}\leftarrow w_{t-1}-\frac{\eta r_{t}}{\left(1-\beta_{1}^{t}\right)u_{t}};$

**16** **end**

### Nesterov adaptive moment estimation (Nadam)

Incorporating Nesterov [28] momentum [29] into Algorithm 4 leads us to a new algorithm, the Nadam algorithm. By using the method of momentum, the learning process is accelerated by summing up the exponential decay of the moving average of the past and current gradients [6]. This method is employed for noisy gradients or gradients with high curvature in particular. Intuitively, the method of momentum combines the opposite signs of gradients in directions of high curvature with higher speed to damp the fluctuations [30]. In the Nesterov method, as a version of the momentum method, we change the step that we evaluate the gradient. In fact, we add a correction factor to the standard method of momentum. All the details are available in Algorithm 6. Lastly, the Nesterov momentum can be applied to the Adamax algorithm to have another variant of Adam, referred to as the Nadamax algorithm [31] [32].

**Algorithm 6:** Nadam

**Input:** Training data *S*, learning rate *η*, weights *w*, fuzz factor *ϵ*, learning rates decay over each update *r*_1_ and *r*_2_, momentum decay rate *γ*, exponential decay rates *β*_1_ and *β*_2_

**Output:** Updated weights *w*

**1** *ϵ* ← *ϵ*_0_ ≈ 10^−8^;

**2** *w* ← *w*_0_;

**3** *t* ← 0;

**4** *r*_1_ ← 0

**5** *r*_2_ ← 0

**6** **while** *stopping criterion is not met* **do**

**7**  Randomly shuffle the training data *S*;

**8**  Sample a minibatch of size *m*:{(*x*^(1)^, *y*^(1)^), …, (*x*^(*m*)^, *y*^(*m*)^)} ∈ *S*;

**9**  **for** *i* ∈ {1, …, *m*} **do**

**10**   ${\hat{G}}_{t}\leftarrow \frac{\partial }{\partial w_{i}}cost\left(w_{t},\left(x^{\left(i\right)},y^{\left(i\right)}\right)\right);$ Gradient calculation

**11**   *t* ← *t* + 1;

**12**  **end**

**13**  ${r_{1}}_{t}\leftarrow \beta_{1}{r_{1}}_{t-1}+\left(1-\beta_{1}\right){\hat{G}}_{t};$;

**14**  ${\hat{r_{1}}}_{t}\leftarrow \frac{{r_{1}}_{t}}{1-\beta_{1}^{t}};$

**15**  $w_{t+1}\leftarrow w_{t}-\frac{\eta }{\epsilon +\sqrt{{r_{2}}_{t}}}\left(\beta_{1}{r_{1}}_{t}+\frac{1-\beta_{1}}{1-\beta_{1}^{t}}{\hat{G}}_{t}\right);$

**16** **end**

### Root mean square propagation (RMSprop)

The RMSprop algorithm [30] is a modified version of the Adagrad algorithm that divides the learning rate by an exponentially decaying average of squared gradients. This step is still similar to Algorithm 3 as depicted in Algorithm 7. RMSprop would outperform Adagrad in the non-convex problems due to the learning rate shrinkage of the Adagrad algorithm as it is explained in Algorithm 2. There is a fancy but expensive implementation of the RMSprop algorithm which calculates the diagonal Hessian which costs double the time of the basic algorithm SGD [18]. This algorithm has shown notable performance in training of deep neural networks and especially in recurrent neural networks [30].

**Algorithm 7:** RMSprop

**Input:** Training data *S*, learning rate *η*, weights *w*, decay rate *ρ*, fuzz factor *ϵ*, learning rate decay over each update *r*

**Output:** Updated weights *w*

**1** *ρ* ← *ρ*_0_;

**2** *ϵ* ← *ϵ*_0_ ≈ 10^−8^;

**3** *w* ← *w*_0_;

**4** *r* ← 0

**5** **while** *stopping criterion is not met* **do**

**6**  Randomly shuffle the training data *S*;

**7**  Sample a minibatch of size *m*:{(*x*^(1)^, *y*^(1)^), …, (*x*^(*m*)^, *y*^(*m*)^)} ∈ *S*;

**8**  **for** *i* ∈ {1, …, *m*} **do**

**9**   $\hat{G}\leftarrow \frac{\partial }{\partial w_{i}}cost\left(w,\left(x^{\left(i\right)},y^{\left(i\right)}\right)\right);$ Gradient calculation

**10**  **end**

**11**  $r\leftarrow \rho r+\left(1-\rho \right)\hat{G}⊙\hat{G};$

**12**  $w\leftarrow w-\frac{\eta }{\sqrt{\epsilon +r}}⊙\hat{G}$

**13** **end**

As shown in Fig 1, once the topology of the network is designed, it is the time to optimize the hyper-parameters of the optimization algorithms such as learning rates. Table 3 presents the parameters setting for the grid-search stage 2. In fact, the optimized number of layers and neurons to construct the deep model architecture were employed to optimize the learning rates for each of the optimization algorithms. Table 4 presents the tuned hyper-parameters that considered for the optimization algorithms. As seen, at this stage the grid-search was done for the list of learning rates and optimization algorithms.

**Table 3:** Parameters setting for the grid-search stage 2.

Hyper-parameter	Settings
Number of layers	8
Number of neurons in each layer	8, 30, 25, 20, 12, 8, 4, 1
Batch sizes	50
Number of epochs	1000
Activation functions	ReLU, Softmax, Sigmoid
Optimizers	SGD, Adagrad, Adadelta, RMSprop, Adam, Adamax, Nadam
Learning rates list	1.0, 0.1, 0.005, 0.002, 0.001
Losses	MSE
Score metrics	R
Number of HPC nodes	10
Number of folds in cross-validation	10

**Table 4:** Optimized hyper-parameters for the optimization algorithms after the grid-search stage 2.

Optimizers	*η*	*ϵ*	*ρ*	*r*	*β*_1_	*β*_2_
SGD	0.01	None	None	0.0	None	None
Adagrad	0.01	1e-08	None	0.0	None	None
Adadelta	1.0	1e-08	0.95	0.0	None	None
RMSprop	0.001	1e-08	0.9	0.0	None	None
Adam	0.001	1e-08	None	0.0	0.9	0.999
Adamax	0.002	1e-08	None	0.0	0.9	0.999
Nadam	0.002	1e-08	None	0.004	0.9	0.999

## Case study

**Fig 3:**
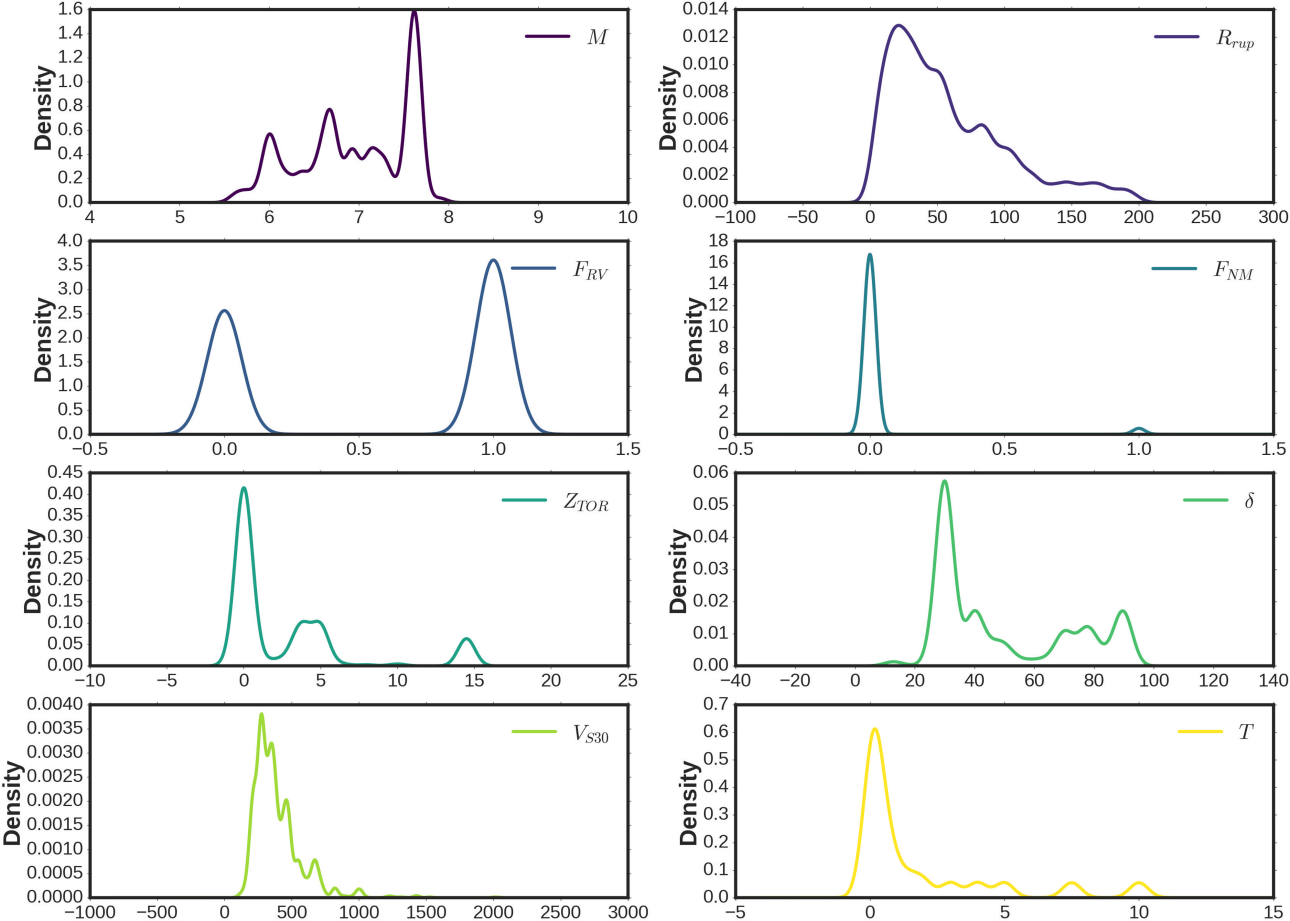
Density plots of the predictor input variables.

**Fig 4:**
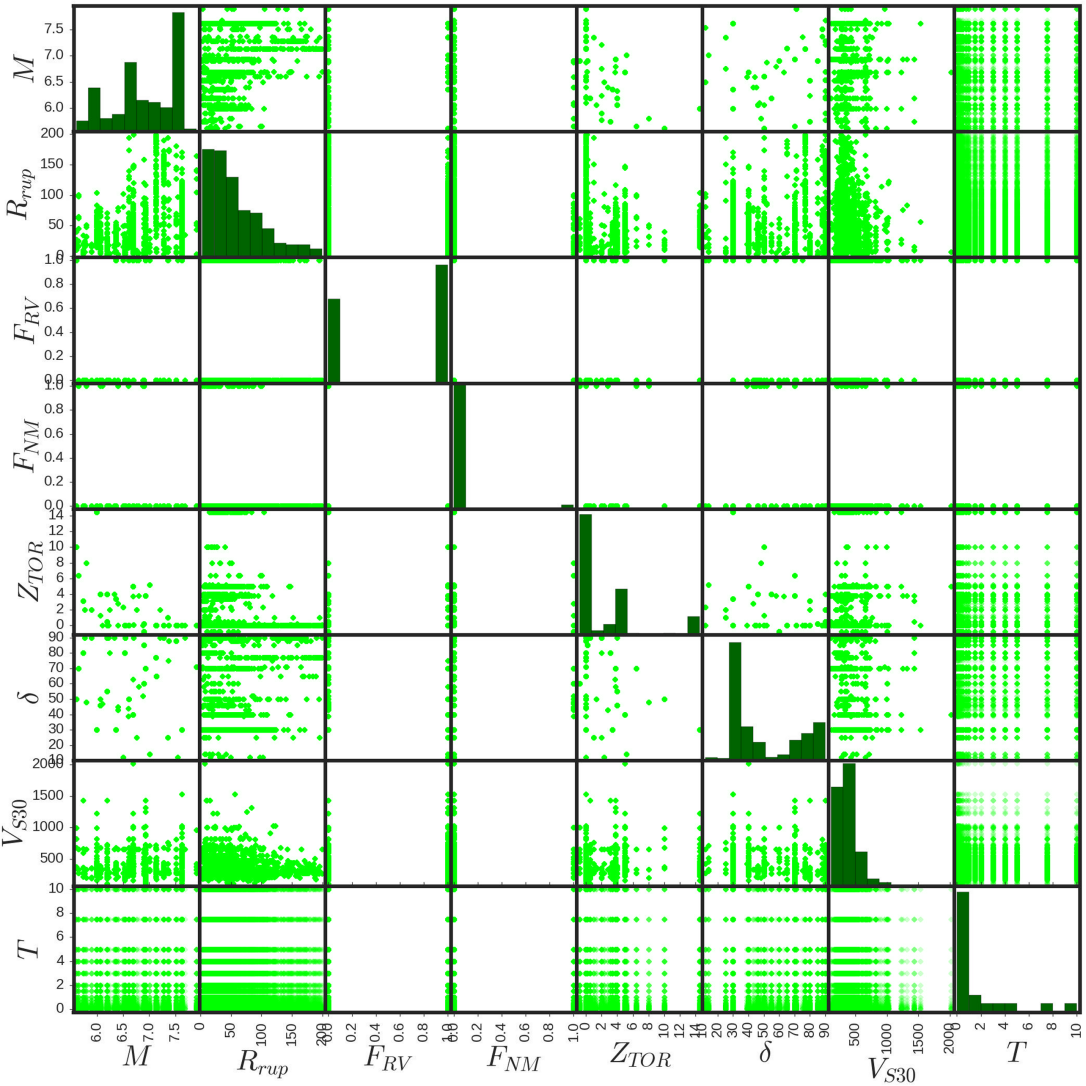
Scatter matrix presentation of the predictor input variables with their probability histograms.

In this paper, a powerful multi-stage deep model using iDeepLe pipeline is proposed for purposes of seismic hazards assessment based on the database (https://ngawest2.berkeley.edu) of the NGA project presented by [11]. The database contains eight feature variables and one response variable including 25,748 exemplars. The proposed model explicitly includes the effects of moment magnitude (*M*), closest distance to the coseismic rupture plane in kilometers (*R*_*rup*_), reverse and reverse-oblique faulting indicator based on the measurements of the average angle of the slip in the plane of rupture between the strike direction and the slip vector (*F*_*RV*_), normal and normal-oblique faulting indicator (*F*_*NM*_), depth to the top of the coseismic rupture plane in kilometers (*Z*_*TOR*_), large rupture dips (*δ*), the time-averaged shear-wave velocity in the top 30 meters of the site profile in meters per seconds (*V*_*S*30_), and periods in seconds (*T*) [11]. The illustration of the density plots of the predictor input variables are depicted in Fig 3. In addition to this, Fig 4 shows the scatter matrix presentation of the predictor input variables with their probability histograms.

By employing the aforementioned predictor variables, a model is desired to measure an approximation of the maximum acceleration that a building is experiencing during an earthquake. This is spectral acceleration (*SA*). *SA* is the maximum acceleration of a damped, single-degree-of-freedom harmonic oscillator, measured in unit of gravity (*g*) specified by two main terms: 1) spectral period, 2) spectral damping. The spectral period is the natural period of the harmonic oscillator in seconds and spectral damping is the degree of damping that we consider for the harmonic oscillator which is usually around 5%. *SA* is usually calculated based on a simple harmonic oscillator simulation made by a particle on a massless vertical rod having the same natural period of vibration as the building [33]. In this paper, the normalized *Ln*(*SA*) is employed as the output of the deep model for the regression task.

## Results & discussion

To test out the performance of the tuned deep model architecture along with tuned hyper-parameters for the optimization algorithms, the grid-search stage 3 with 10-folds cross-validation for four different batch sizes over seven different number of epochs was applied. In this stage two loss metrics including mean-squared-error (*MSE*) and mean-absolute-error (*MAE*) and two score metrics including coefficient of determination (*R*^2^), and explained variance (*EV*) are considered. The parameters setting for the grid-search stage 3 is presented in Table 5. In addition to this, the tuned hyper-parameters involved in optimization algorithms are also presented in Table 4.

**Table 5:** Parameters setting for the grid-search stage 3.

Hyper-parameter	Settings
Number of layers	8
Number of neurons in each layer	8, 30, 25, 20, 12, 8, 4, 1
Batch sizes	50, 100, 150, 200
Number of epochs	50, 100, 200, 300, 500, 800, 1000
Activation functions	ReLU, Softmax, Sigmoid
Optimizers	SGD, Adagrad, Adadelta, RMSprop, Adam, Adamax, Nadam
Learning rates	0.01, 0.01, 1.0, 0.001, 0.001, 0.002, 0.002
Losses	MAE, MSE
Score metrics	R, EV
Number of HPC nodes	10
Number of folds in cross-validation	10

**Fig 5:**
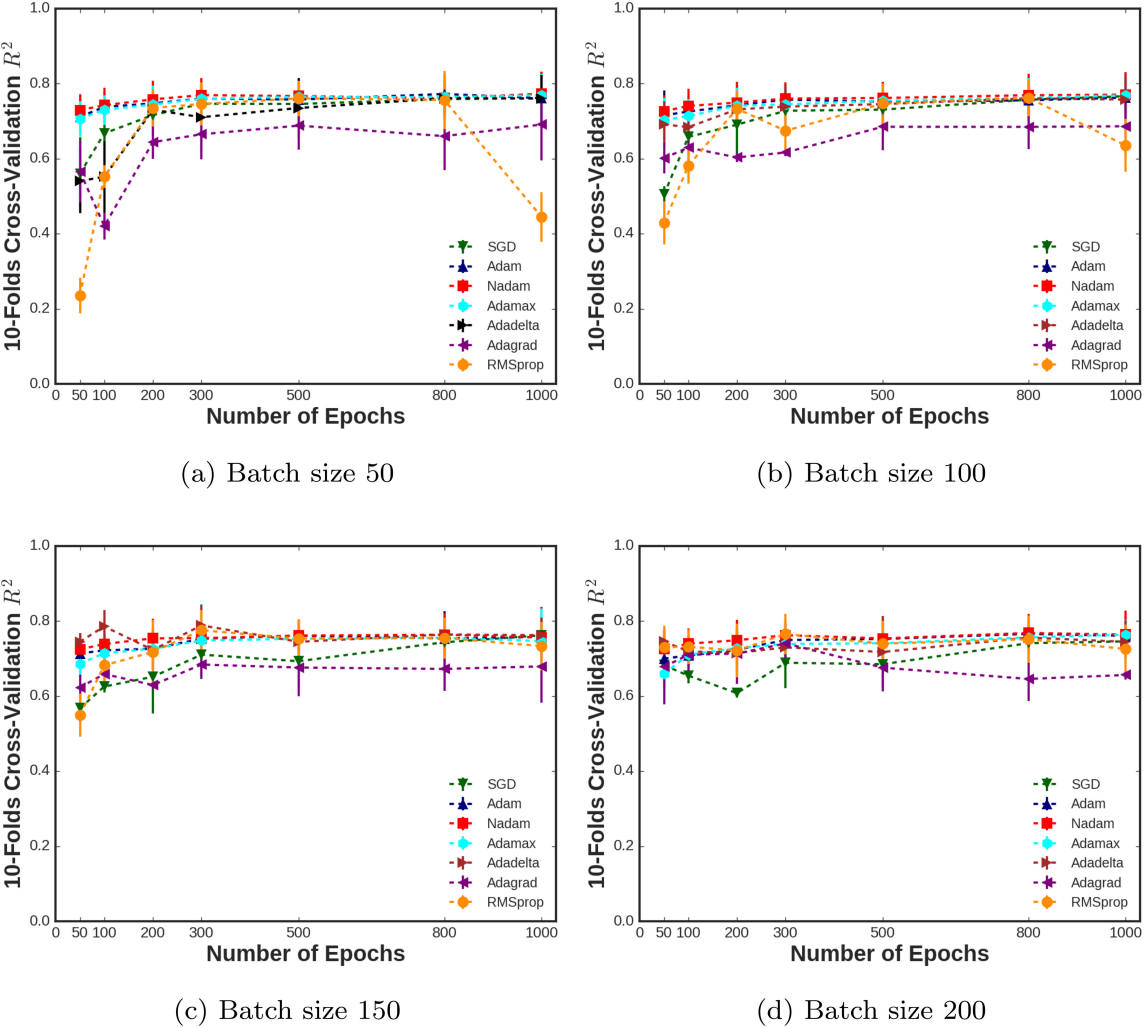
10-folds cross-validation *R*^2^ during number of epochs for different batch sizes employing different optimization algorithms.

As the first task, 10-folds cross-validation of the coefficient of determination (*R*^2^) with its standard deviation through a different number of epochs for different batch sizes (50, 100, 150, and 200) are presented in Fig 5, respectively. These figures show the performance of the deep model in terms of the proportion of the prediction variance which vary for different configurations (i.e. optimizers, epochs, and batch sizes). As seen here, as we increased the number of epochs, we can see the convergence of the algorithms. Although the model converged after 300 iterations, it is necessary to test out the performance of the model for a large number of epochs to prove the stability of the deep model. It is interesting that RMSprop scores decayed after 800 epochs in Fig 5(a) and 5(b). This may be due to the lack of bias-correction, which reducing the RMSprop’s performance towards the end of optimization as gradients become sparser. In addition to this, the better performance of Nadam might be due to applying moving average before rescaling gradient. However, this procedure in RMSprop is exactly in different order which is another reason to explain RMSprop performance. It is also obvious that for a low number of batch sizes such as 50 and 100, the variation of the scores are higher before the algorithms get to the convergence points. This suggests that for the low number of epochs, it would be better to employ larger batch sizes. It is also clear that Nadam outperformed the other algorithms with higher scores and lower fluctuations. This could be due to the presence of Nesterov momentum [28] as discussed in Algorithm 6.

As the second task, in order to see the performance of the optimization algorithms for the selected database, two loss metrics, *MAE* and *MSE* through different batch sizes were tested out. In addition, two score metrics, *R*, and explained variance (*EV*) were also presented for different batch sizes [34]. The explained variance measures the proportion to which the proposed model accounts for the variance of the selected database in terms of prediction of spectral acceleration.

**Fig 6:**
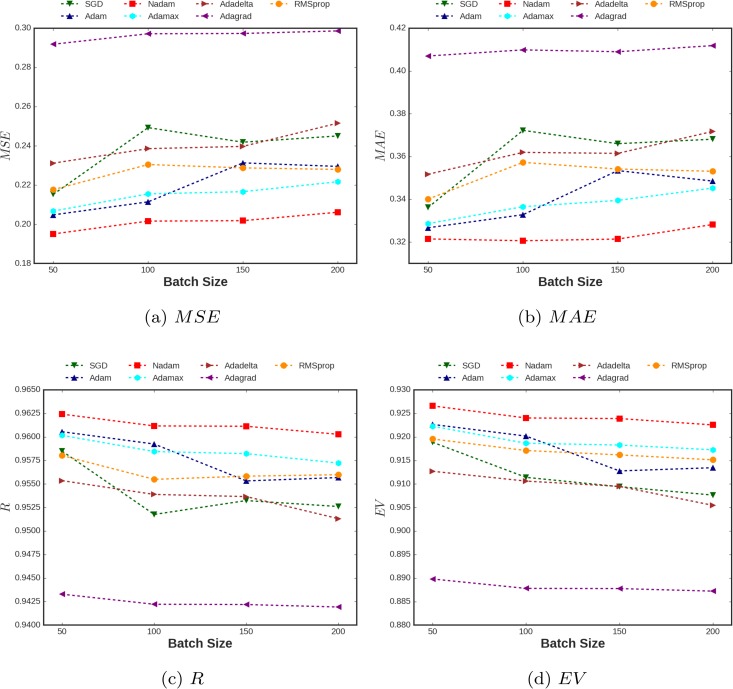
Statistical metrics for different batch sizes employing different optimization algorithms.

As seen in Fig 6(a) and 6(b), as we increased the batch sizes, the losses increased as was expected. It should also be noted that there are some exceptions for RMSprop and SGD algorithms that the loss metrics decrease for batch size 150. Fig 6 can be a perfect illustration to rank the performance of the optimization algorithms. Obviously, variants of Adam algorithm, specifically Nadam, outperformed the other algorithms. The worst performance has been seen for the Adagrad algorithm. It also should be considered that the deviation of *R* score as a metric for the regression task is just around 2% between the best (0.96264) and the worst (0.94246) algorithms. The closest explained variance to 1.0 is presented using the Nadam algorithm which showed the best performance among the optimizations algorithms for this selected database.

**Fig 7:**
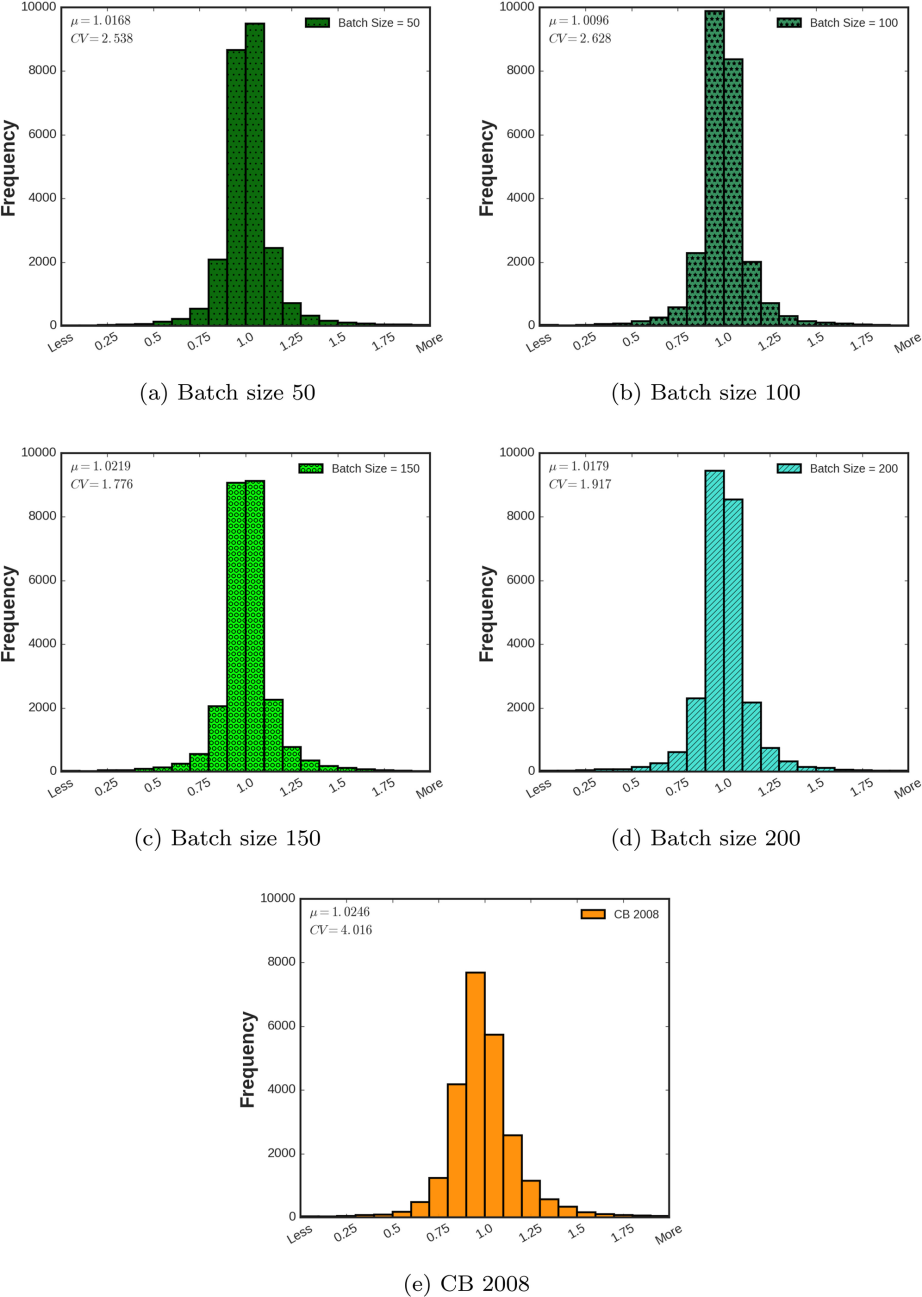
Histograms of the ratio of the predicted and the measured *Ln*(*SA*) for different batch sizes. Mean and coefficient of variation of this ratio are also reported for different batch sizes. CB 2008 model has also been demonstrated for comparison.

One of the most well-known models for modeling of particles motions during earthquake is proposed by [11] as a part of NGA project. This model is called “CB 2008” and it is used to benchmark the proposed deep models in this study. Fig 7 presents the histograms of the ratio of the predicted and the measured *Ln*(*SA*) for different batch sizes and the CB 2008 model. In order to study the quality assurance, mean and coefficient of variation (CV) [34] of this ratio are also reported for different batch sizes. As shown, the mean values of all the histograms are approximately equal to one. The histogram with batch size 100 presents the best mean value of 1.0096. More interesting is the coefficient of variation of the presented histograms and the difference between iDeepLe results and the CB 2008 model. The coefficient of variation of the CB 2008 model is about two times more than the coefficients of variation of the proposed models. Employing coefficient of variation of a model helps to have better understanding of standard deviation of data in the context of the mean value of the data. As it is shown in Fig 7(e), the ratio of the predicted and measured spectral acceleration has a normal distribution with a mean value of 1.0246. However, its calculated coefficient of variation indicated that data had a large variation. This phenomenon can also be understood from the maximum frequency value presented in Fig 7(e) which is around 8000. On the other hand, the maximum values in the other histograms are about 10000. This indicated another point of out-performance of the proposed iDeepLe model with respect to the CB 2008 model.

[35] suggested new criteria for external verification of a proposed model: the slope(*K* or *K*′) of the regression line between the actual data (*h*_*i*_) and the predicted data (*t*_*i*_) should be close to 1, and the performance indices |*m*| and |*n*| should be lower than 0.1. Recently, [36] introduced an index (*R*_*m*_) for external predictability evaluation of models. Their validation criterion is satisfied for *R*_*m*_ ≥ 0.5. The external validation of the statistical parameters of the deep models for different batch sizes and CB 2008 model are presented in Table 6. The proposed deep models with the different batch sizes satisfy all the validation conditions. However, CB 2008 model does not satisfy the last two conditions.

**Table 6:** External validation results of the deep models with different batch sizes and CB 2008 model.

	Condition	Batch 50	Batch 100	Batch 150	Batch 200	CB 2008
*R*	*R* ≥ 0.8	0.96264	0.96150	0.96120	0.96031	0.91938
$K=\frac{\sum_{i=1}^{n}h_{i}t_{i}}{h_{i}^{2}}$	0.85 < *K* < 1.15	0.99710	0.98845	0.99373	0.98863	0.96735
$K^{\prime }=\frac{\sum_{i=1}^{n}h_{i}t_{i}}{t_{i}^{2}}$	0.85 < *K*′ < 1.15	0.99332	1.00164	0.99632	1.00123	1.01279
$R_{m}=R^{2}\left(1-\sqrt{|R^{2}-R_{0}^{2}|}\right)$	*R*_*m*_ ≥ 0.5	0.67588	0.67220	0.66959	0.66673	0.52372
$R_{o}^{2}=1-\frac{\sum_{i=1}^{n}{\left(t_{i}-h_{i}^{0}\right)}^{2}}{\sum_{i=1}^{n}{\left(t_{i}-\bar{t_{i}}\right)}^{2}}$	$h_{i}^{0}=K\times t_{i}$	0.99993	0.99896	0.99967	0.99893	0.98997
$R_{o}^{{}^{\prime }2}=1-\frac{\sum_{i=1}^{n}{\left(h_{i}-t_{i}^{0}\right)}^{2}}{\sum_{i=1}^{n}{\left(h_{i}-\bar{h_{i}}\right)}^{2}}$	$t_{i}^{0}=K^{\prime }\times h_{i}$	0.99966	0.99896	0.99967	0.99893	0.98997
$\left|m\right|=\frac{|R^{2}-R_{0}^{2}|}{R^{2}}$	|*m*| < 0.1	0.07904	0.08056	0.08201	0.08321	0.17120
$\left|n\right|=\frac{|R^{2}-R_{0}^{{}^{\prime }2}|}{R^{2}}$	|*m*| < 0.1	0.07875	0.08166	0.08225	0.08436	0.18158

As shown in this paper, all the variants of the Adam algorithm, specifically Nadam algorithm, had the best performances in terms of both accuracy and computational cost. Therefore, it would be an intelligent decision to employ one of its variants as the optimization algorithm to tune the hyper-parameters involved in deep learning tasks. This would be an appropriate choice of optimization method rather than basic SGD algorithms. This decision can be supported by the vast use of the Adam algorithm in Google DeepMind (https://deepmind.com/).

As explained, the main goal of this study was to point out the possibility of building an optimized neural network from scratch for a database with any size. An alternative way of developing a model from scratch is employing a pre-trained neural network. However, this stage requires vast knowledge of neural network modeling to modify the model to match the dimension of the data and show reasonable performance. Table 7 is presented in order to show how close the presented tuned hyper-parameters using Adam algorithm are to the default values of Adam in the most popular deep learning libraries.

**Table 7:** Parameters setting for the Adam optimizer using the popular deep learning libraries.

Libraries	*η*	*ϵ*	*β*_1_	*β*_2_
Keras	0.001	1e-08	0.9	0.999
TensorFlow	0.001	1e-08	0.9	0.999
Caffe	0.001	1e-08	0.9	0.999
Lasagne	0.001	1e-08	0.9	0.999
Torch	0.001	1e-08	0.9	0.999
MxNet	0.001	1e-08	0.9	0.999
Blocks	0.002	1e-08	0.9	0.999

## Comparative study

**Fig 8:**
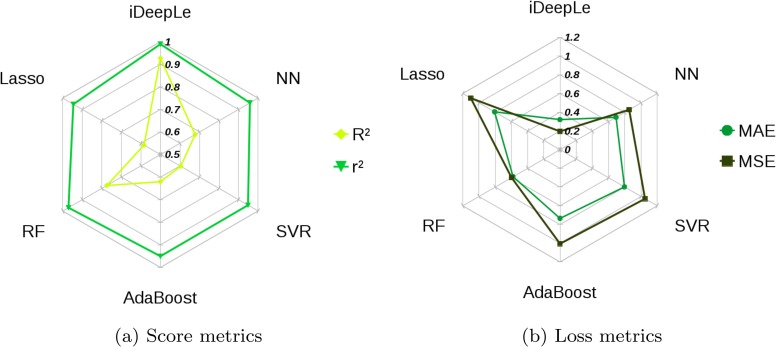
Radar plots of the regression metrics for the most common regression models compared to iDeepLe model.

As the final assessment of this study, the performance of the most common regression models including: (1) least absolute shrinkage and selection operator (Lasso), (2) random forest (RF), (3) adaptive boosting (AdaBoost), (4) support vector regression (SVR), and (5) multilayer perceptron neural network (NN) were compared to iDeepLe model employing 10-folds cross-validation. Fig 8 illustrates the radar plot of the performance of the regression models in terms of *R*^2^ and *r*^2^, (shown in Fig 8(a)), and *MAE* and *MSE* (shown in Fig 8(b)). By comparing the coefficient of determination, it is clear that iDeepLe with *R*^2^ of 0.92 has the best performance and the closest *R*^2^ to the proposed model is RF with *R*^2^ of 0.77. However, all the models have high *r*^2^ values and iDeepLe still has the highest one. In addition to this, RF after iDeepLe has the lowest *MAE* with 0.57 and *MSE* with 0.59 but they are still far more than the iDeepLe’s. It should be noted that Lasso showed the worst performance among the employed regression models for the selected database.

## Conclusions

This paper proposes a powerful multi-stage deep learning pipeline to formulate the spectral acceleration based on the ground-motion predictor variables for the purposes of seismic hazards assessment. In the first stage, it is desired to optimize the topology of the network to find the number of layers, the number of neurons in each layer, and the type of activation function for each layer. In the second stage, the optimization of the learning rates for each of the optimization algorithms is desired. In the third stage, the performance of the model with the optimized topology and learning rates is tested with different number of epochs and batch sizes for different optimization algorithms. In order to optimize the hyper-parameters, seven optimization algorithms including modern algorithms with adaptive learning rates were employed. The pseudo codes, pros and cons of each algorithm are discussed briefly. The developed deep model can automatically select the most significant predictor input variables, formulate the model structure, and solve the unknown parameters of the regression equation. The performance of the proposed model was compared to the most famous model thus far (CB 2008), for the selected database [11]. Based on the results, the following conclusions are drawn:

1. The multi-stage nature of the proposed model along with adaptive learning rates optimization algorithms increases the regression accuracy and decreases the cost and the complexity.
2. The proposed deep model correlates the predictor input data with spectral acceleration for 96.26% which outperforms CB 2008 model with an accuracy of 91.93% as well as popular regression models including Lasso, RF, AdaBoost, SVR, and NN.
3. The results show that the Nadam algorithm has the most significant performance in the proposed model. On the other hand, the Adagrad algorithm has the poorest performance in comparison to the other algorithms for in the deep model respectively.
4. The proposed iDeepLe along with the Nadam algorithm based on its parallel structure is shown to be a fast enough tool that it can be employed to generate solid and accurate models for complex non-linear systems.
5. The statistical parameters presented in Table 6 validate the proposed deep model which outperforms the previous presented results based on the same database.
6. Adam algorithm and its variants combine the best properties of the Adagrad and RMSProp algorithms to provide an optimization algorithm that can handle sparse gradients on noisy problems. Thus, this algorithm would be an appropriate choice for benchmarks in deep learning studies.

Although, prediction of spectral acceleration experienced by a particle during earthquakes is a very challenging problem, the use of the proposed multi-stage deep model is not only limited to this problem and can be employed in various applications. Therefore, the future research could be developing iDeepLe model for other challenging problems.

## Appendix

### Appendix A. Regression metrics

- $MAE=\frac{\sum_{i=1}^{n}\left|h_{i}-t_{i}\right|}{n}$
- $MSE=\frac{\sum_{i=1}^{n}{\left(h_{i}-t_{i}\right)}^{2}}{n}$
- $R^{2}={\left[\frac{\sum_{i=1}^{n}\left(h_{i}-\bar{h_{i}}\right)\left(t_{i}-\bar{t_{i}}\right)}{\sqrt{\sum_{i=1}^{n}{\left(h_{i}-\bar{h_{i}}\right)}^{2}\sum_{i=1}^{n}{\left(t_{i}-\bar{t_{i}}\right)}^{2}}}\right]}^{2}$
- $r^{2}=\frac{\sum_{i=1}^{n}h_{i}^{2}-\sum_{i=1}^{n}{\left(h_{i}-t_{i}\right)}^{2}}{\sum_{i=1}^{n}h_{i}^{2}}$

where *h*_*i*_ and *t*_*i*_ are respectively the measured and predicted values for the *i*^*th*^ output, $\bar{h_{i}}$ and $\bar{t_{i}}$ are the average of the measured and predicted outputs, and *n* is the total number of instances.

### Appendix B. Regression parameters in comparative study

- Lasso: *ϵ* = 2.22*e* − 16, *N*_*iteraion*_ = 500.
- RF: *N*_*tree*_ = 100.
- AdaBoost: *N*_*stage*_ = 100, *η* = 0.1.
- SVR: *kernel* = *linear*, *tol* = 0.001, *C* = 1.0, *ϵ* = 0.1.
- NN: *N*_*hidden*_ = 100, *F*_*activation*_ = *ReLU*, *OPT*_*algorithm*_ = *adam*, *η* = 0.001, *ϵ* = 1*e* − 08.

where *ϵ* is the machine-precision regularization in the computation of the Cholesky diagonal factors, *N*_*iteration*_ is the maximum number of iterations, *N*_*tree*_ is the number of trees, *N*_*stage*_ is the number of boosting stages to perform, *η* is learning rate, *tol* is the tolerance for stopping criterion, *C* is the penalty parameter of the error term, *N*_*hidden*_ is number of hidden layers in the network, *F*_*activation*_ is the activation function, and *OPT*_*algorithm*_ is the optimization algorithm to perform.
